# The Unfolded Protein Response Supports Plant Development and Defense as well as Responses to Abiotic Stress

**DOI:** 10.3389/fpls.2017.00344

**Published:** 2017-03-15

**Authors:** Yan Bao, Stephen H. Howell

**Affiliations:** Plant Sciences Institute and the Department of Genetics, Development and Cell Biology, Iowa State University, AmesIA, USA

**Keywords:** protein folding, ER (endoplasmic reticulum) stress, IRE1, bZIP28, regulated-IRE1 dependent RNA decay (RIDD), auxin, brassinosteroid, plant virus

## Abstract

The unfolded protein response (UPR) is a stress response conserved in eukaryotic organisms and activated by the accumulation of misfolded proteins in the endoplasmic reticulum (ER). Adverse environmental conditions disrupt protein folding in the ER and trigger the UPR. Recently, it was found that the UPR can be elicited in the course of plant development and defense. During vegetative plant development, the UPR is involved in normal root growth and development, the effect of which can be largely attributed to the influence of the UPR on plant hormone biology. The UPR also functions in plant reproductive development by protecting male gametophyte development from heat stress. In terms of defense, the UPR has been implicated in virus and microbial defense. Viral defense represents a double edge sword in that various virus infections activate the UPR, however, in a number of cases, the UPR actually supports viral infections. The UPR also plays a role in plant immunity to bacterial infections, again through the action of plant hormones in regulating basal immunity responses.

## Introduction

The unfolded protein response (UPR) is widely regarded as a stress response which is activated by stress conditions in the endoplasmic reticulum (ER) (Hartl and Hayer-Hartl, 2009; Gardner et al., 2013). ER stress is brought about by a variety of different conditions that can lead to the accumulation of misfolded or unfolded proteins in the ER (Duwi Fanata et al., 2013). These conditions include abiotic stresses, such as high temperature, salt stress or biotic agents, such as viral or bacterial pathogens. The UPR is also activated under protein synthesis overload conditions when the need for protein folding simply cannot meet demands (Liu and Howell, 2010b, 2016; Kørner et al., 2015).

**FIGURE 1:**
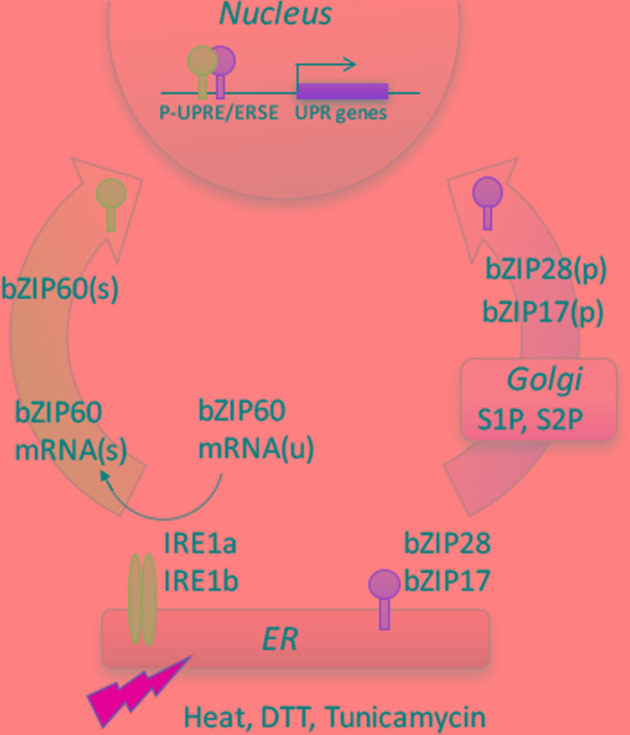
**Two branches of UPR signaling pathway in plants.** One branch involves the dual protein kinase and ribonuclease, IRE1, which splices bZIP60 mRNA when activated. The other branch is mediated by two ER membrane-anchored transcription factors, bZIP17 and bZIP28. Different stresses interfere with protein folding in the ER, leading to an accumulation of unfolded or misfolded proteins in the ER, activating the UPR. The splicing of bZIP60(u) mRNA introduces a frameshift, such that the resultant spliced form bZIP60(s) mRNA is translated into a transcription factor targeted to the nucleus. ER stress also provokes the mobilization of the membrane-anchored transcription factors from ER to Golgi, where they are processed to bZIP17(p) and bZIP28(p) by Golgi resident S1P and S2P proteases, releasing their cytosolic transcription factor domains. The factors from both branches are targeted to the nucleus, and either homodimerize or heterodimerize to bind to the promoters and regulate the expression of stress response genes. Based on Howell (2013b).

Stress conditions in the ER are communicated to the nucleus via the UPR signaling pathway. There are two arms to this pathway in plants (Howell, 2013a) (**Figure 1**). One arm is mediated by RNA splicing factor, IRE1, an ER transmembrane protein with its N-terminus facing the ER lumen and its C-terminus, bearing both its protein kinase and ribonuclease domains, facing the cytosol. The lumenal domain of IRE1 senses the protein status in the ER. The primary target of IRE1 in plants is bZIP60 mRNA which is spliced in response to stress (Deng et al., 2011). In *Arabidopsis*, IRE1’s cleavage of bZIP60 mRNA results in the excision of a 23 base-pair intron (Deng et al., 2011; Nagashima et al., 2011). The unspliced form of bZIP60 mRNA encodes a membrane-anchored transcription factor, however, splicing causes a frame shift eliminating the transmembrane domain, yielding a form of bZIP60 (bZIP60s) targeted to the nucleus (Deng et al., 2011). Under normal growth conditions, the unspliced form of bZIP60 (bZIP60u) is transcribed and translated (Iwata et al., 2008; Nagashima et al., 2011), but it is not yet clear what its function might be.

Beside its splicing function, IRE1 also attacks other mRNAs in response to stress in a process called regulated-IRE1 dependent RNA decay (RIDD) (Hollien and Weissman, 2006; Hollien et al., 2009). Studies by Mishiba et al. (2013) showed that RIDD in *Arabidopsis* largely targets mRNAs encoding secretory pathway proteins. Thus, IRE1 is a major factor shaping the stress transcriptome, upregulating genes by promoting the production of a potent transcription factor (bZIP60s) and by degrading other transcripts through RIDD (Mishiba et al., 2013).

The other arm of the ER stress signaling pathway is mediated by ER membrane-associated transcription factors, bZIP17 and bZIP28 (**Figure 1**). Under unstressed conditions, these factors are retained in the ER by their association with binding protein (BiP). In response to stress, when misfolded proteins accumulate in the ER, BiP is competed away and disassociates from bZIP28 (Srivastava et al., 2013). Once liberated, the factors relocate from the ER to the Golgi, where they are cleaved by two Golgi-resident proteases, Site-1 and Site-2 protease (S1P and S2P) (Liu et al., 2007a; Che et al., 2010). Cleavage by S2P in the Golgi membrane releases the cytosolic-facing components of bZIP17/bZIP28 from the Golgi allowing for their transport into the nucleus to upregulate the expression of stress response genes (Liu et al., 2007a,b; Liu and Howell, 2010a).

## The UPR in Vegetative Development

The UPR has been extensively studied in the context of ER stress, although recently more attention has been paid to the role of the UPR in plant development and defense. The UPR has been found to play roles in both vegetative and reproductive development. Vegetative development studies have focused on root development, and under normal growth conditions, root growth is inhibited in *ire1a ire1b* double mutants that knock out both IRE1 isoforms in *Arabidopsis* (Chen and Brandizzi, 2012). *IRE1a* and *IRE1b* have overlapping functions, however, the extent of overlap has not been fully resolved (Nagashima et al., 2011; Chen and Brandizzi, 2013; Howell, 2013b). In some studies *IRE1b* appears to be more active in response to ER stress agents (Deng et al., 2011),while IRE1a is reported to play a more prominent role in certain biotic stress responses (Moreno et al., 2012).

**FIGURE 2:**
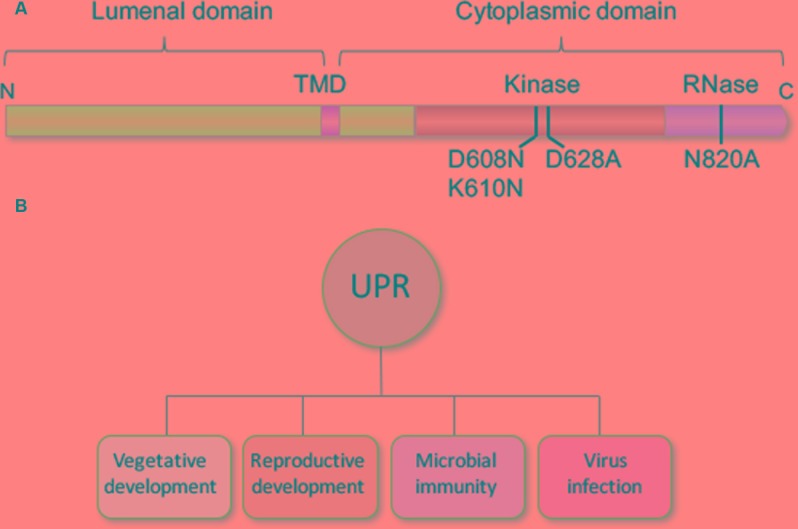
**Structural map of IRE1b and diverse roles for the UPR.** **(A)** IRE1b has a single transmembrane domain (TMD) and localizes in the ER membrane with its N-terminus in the ER lumen and its C-terminus facing the cytosol. Numbers below the diagram represent residues, which when mutated specifically block the following activities: D608N, K610N block the nucleotide binding activity, D628A knocks out the protein kinase catalytic activity and D820A interferes with the RNase activity (Deng et al., 2013). **(B)** The UPR plays roles beyond stress to include vegetative and reproductive development, microbial immunity, and virus infection.

IRE1 is a multifunctional protein with ribonuclease and protein kinase domains, and IRE1b has been dissected with site-specific mutations in an effort to learn which of its functional domains is required for normal root growth (**Figure 2A**). One of the site-specific mutations knocks out the ribonuclease activity of IRE1b (N820A) while two other affect activities associated with the protein kinase domain (Deng et al., 2013). Of the latter two, one blocks the catalytic activity of the protein kinase (D628A), while the other double mutant prevents nucleotide binding (D608N, K610N), which is required for activating the ribonuclease activity of IRE1b. In complementation experiments with these site-specific IRE1b mutations, it was found that neither the mutation in the ribonuclease domain (N820A) or the nucleotide binding domain (D608N, K610N) could restore normal root growth in an *ire1a ire1b* double mutant (Deng et al., 2013). However, the mutation in catalytic site of the protein kinase domain (D628A) complemented root growth in the *ire1a ire1b* mutant, meaning that the ribonuclease function of IRE1 is necessary for normal root growth, but that the catalytic activity of IRE1’s protein kinase is dispensable.

The principal target of IRE1’s RNA splicing activity is bZIP60 mRNA, and so it is curious that the short root phenotype is not observed in *bzip60* single or *bzip28 bzip60* and *bzip17 bzip60* double mutants. This finding argues that the impact of IRE1 on root development is dependent on the ribonuclease activity of IRE1, but independent of its principal RNA splicing target bZIP60 mRNA (Deng et al., 2013). The only ribonuclease activity of IRE1 that is known to be independent of bZIP60 is its RIDD activity, the promiscuous ribonuclease activity of IRE1 in attacking other mRNAs encoding secretory proteins (Mishiba et al., 2013). Interestingly, the short root phenotype of *ire1a ire1b* is also observed in *bzip28 ire1b* seedlings, implying that the two branches of the UPR signaling pathway coordinately influence root development (Deng et al., 2013).

## The UPR in Plant Hormone Biology

The UPR is reported to play an unsuspected role in hormone biology, which may explain the influence of the UPR on vegetative growth and development. Chen et al. (2014) recently examined the possible relationship between the UPR and auxin regulation in *Arabidopsis*. They found, quite unexpectedly, that ER stress down-regulates the expression of genes encoding ER- and PM-localized auxin efflux transporters (PIN-formed or PIN proteins) and the auxin receptors TIR1/AFBs. The extent of down-regulation was modest, and the mechanism by which this happens is unclear. It does not require IRE1, which eliminates the possibility that RIDD might be involved in the down-regulation. One consequence of the down-regulation of auxin receptors TIR1/AFBs was the possible stabilization of AUX/IAA proteins. Chen et al. (2014) analyzed the levels DII-VENUS, a fluorescently tagged AUX/IAA surrogate that contains the degron responsible for auxin-induced TIR1/AFB-mediated protein degradation. They found that the levels of DII-VENUS increased in response to ER stress suggesting that under stress, auxin responsive genes may remain repressed (or otherwise controlled) by AUX/IAA in the presence of auxin.

On the other hand, Chen et al. (2014) also reported that activation of UPR requires certain auxin regulators and ER-localized PINs, such as PIN5 and PIN6. They found that in response to ER stress, loss-of-function *pin5*, *pin6* and even *abf1* mutants showed reduced expression of some common UPR biomarker genes, such as *BIP1* and *2* and *PROTEIN DISULFIDE ISOMERASE6* (*PDI6*). Again, the effect was modest and there was no clear explanation for this phenomenon, although the authors speculated that the mutants might affect organellar distribution of free auxin, which might influence ER stress responses in some undefined way.

The UPR has also been implicated in brassinosteroid (BR)-mediated responses. The relationship appears to involve the membrane-associated transcription factors, bZIP17 and bZIP28, and not the RNA splicing arm of the UPR signaling pathway. The relationship was uncovered in mutants of *S2P*, a gene encoding a Golgi-resident protease that processes bZIP17 and bZIP28 when mobilized by ER stress (Che et al., 2010). Mutants in *S2P* have a short root phenotype, which can be overcome by expressing a preprocessed form of bZIP17 (bZIP17ΔC) or bZIP28 (bZIP28ΔC). The relationship of this phenomenon to BR signaling derives from the fact that high levels of a BR agonist, brassinolide (BL), inhibit root growth in WT seedlings, but not in *s2p* mutants and that the root growth inhibition by BL in WT can be overcome by expressing bZIP17ΔC or bZIP28ΔC.

Che et al. (2010) also found that bZIP17ΔC or bZIP28ΔC assists in activating BR responses in *bri1-5* mutants. The BR receptor in *bri1-5* mutants is functional, but defective in trafficking to the cell surface. Thus, the expression of bZIP17ΔC or bZIP28ΔC likely aids the BR receptor in *bri1-5* in trafficking, but not in other aspects of BR signaling. That notion was reinforced by the finding that bZIP17ΔC or bZIP28ΔC expression did not rescue *bri1-6* and *det2* mutants, defective respectively in BR perception and synthesis. Nonetheless, to show that bZIP17ΔC or bZIP28ΔC expression helped to make the BR receptor in *bri1-5* operational, the authors demonstrated that the expression of either of the two bZIPΔCs partially restored BES1 dephosphorylation (a measure of BR signaling by the BRI1 receptor) and the upregulation of BR-induced genes in response to BL.

## The UPR in Reproductive Development

Recently, it was shown that the UPR also plays a role in protecting plant reproductive development from elevated temperature. Plants are vulnerable to heat stress during the reproductive phase in their life cycle, and Deng et al. (2016) showed the RNA splicing arm of the UPR guides *Arabidopsis* reproductive development in such a way so as to protect it from elevated temperature. The authors found that the double *ire1a ire1b* mutant and a mutant in the immediate downstream target, bZIP60, were sterile at elevated temperature. Through, reciprocal crosses it was revealed that the temperature sensitive sterility was a male trait and impacted pollen production. The defect was also found to be sporophytic in nature, and at elevated temperature it affected the structure of the tapetum, which is ER-rich nurse tissue for the developing male gametophyte. The tapetum contributes materials for the formation of the pollen wall and coat, and it was observed that the defect dramatically affected the proper deposition of the pollen coat.

In another study, Deng et al. (2013) dissected IRE1b as described in the previous section with the intent of finding out what IRE1 activities protect male gametophyte development. Using site-specific IRE1b mutants in complementation experiments, the authors demonstrated that, both kinase and RNase functions of IRE1 are required to promote and protect male reproductive development. Deng et al. (2016) further found that bZIP60, the downstream target of IRE1, is also required for temperature protection of male gametophyte production. IRE1 splices bZIP60 mRNA to make an active transcription factor, and there were major expression changes in genes that likely contribute to pollen wall/coat construction, such as small cysteine rich pollen coat proteins. Quite surprisingly, the expression of SEC31A, a protein involved in COPII vesicle formation, restored fertility of *ire1a ire1b* at elevated temperature. *SEC31A* is the gene most highly dependent on *IRE1a IRE1b* function during ER stress in vegetative tissue. The basis for *SEC31A’s* fertility restoration activity in *ire1a ire1b* mutants at elevated temperature is not known, but it is speculated that its contribution to ER to Golgi trafficking may compensate for other defects in the double mutant (Deng et al., 2016).

The role of the UPR in normal plant development was unsuspected because the UPR is thought to be quiescent under normal conditions and is only activated by stress. Does the unspliced form of bZIP60 produced under normal conditions, but upregulated in response to stress, have some function that we are not aware of? On the other hand, the UPR may have a low level of activity under “normal” conditions, sufficient to play a supporting role in plant development. What activates IRE1 under these conditions is not known, although it is speculated that the heavy demand for protein synthesis and/or secretion during development may activate the UPR.

## The UPR and Microbial Immunity

The UPR is also reported to play roles in bacterial immunity. Tateda et al. (2008) found that when *Nicotiana benthamiana* was inoculated with a non-host pathogen, *Pseudomonas cichorii*, and a host-specific pathogen, *Pseudomonas syringae*, the non-host pathogen led to the upregulation in expression of bZIP60, while the host-specific pathogen did not. The authors silenced *N. benthamiana bZIP60* using virus induced gene silencing (VIGS) and found that the plants became more susceptible to the non-host pathogen. Therefore, the authors conclude that the UPR, and more specifically the expression of bZIP60, is an important component of immunity to host pathogens.

Likewise, Moreno et al. (2012) reported that the UPR confers bacterial immunity to *Arabidopsis*. In their analysis, they observed that *ire1a ire1b* double mutants and a mutant in their downstream target, *bzip60*, were more susceptible to *P. syringae* avrRpt2. In addition, the mutants were less able to establish systemic acquired resistance (SAR) to the bacteria when treated with salicylic acid (SA), the only plant hormone which is known to induce UPR in *Arabidopsis* (Nagashima et al., 2014). A signature of SAR in *Arabidopsis* is the secretion of Pathogenesis Related Protein 1 (PR1), and low levels of secreted PR1 were found in SA-treated *ire1a ire1b* double mutants and also in the *ire1a* single mutant. From this, Moreno et al. (2012) argued that IRE1-bZIP60 branch of UPR is involved in SA-mediated plant immune responses and that mutants compromised in the UPR are more susceptible to bacterial pathogens.

## The UPR and Virus Infections

The UPR also plays a significant role in plant virus infection and immunity (Ye et al., 2011). However, the role of UPR is a double edge sword in that on the one hand the UPR appears to bolster plant immunity, but on the other hand the UPR assists in virus infection. Tobacco mosaic virus (TMV) and the tobacco N-gene is a classic case of virus resistance in plants, which appears to involve the UPR in strengthening plant immunity. The N-gene was identified and cloned by Dinesh-Kumar et al. (1995) as a resistance R gene. During N-mediated defense, a number of genes characteristic of the UPR are upregulated including protein disulfide isomerases, ERp57 and P5, calreticulin 3, glucose-regulated protein 78 (GRP78) and BiP5 (Caplan et al., 2009). To determine whether the upregulation of these genes in tobacco was of consequence to TMV infection, VIGS was used to suppress their expression. Silencing of these genes did, indeed, result in a loss of virus containment in inoculated leaves but did not fully prevent the programmed cell death caused by the virus (Caplan et al., 2009). Thus, this observation implicates the UPR in bolstering N-mediated TMV immunity.

However, there are far more examples for how the UPR supports viral infection. ER membrane expansion is an integral part of the UPR, and *Brome mosaic virus* (BMV), *Tobacco etch virus* (TEV), *Cowpea mosaic virus* (CPMV), *Red clover necrotic mosaic virus* (RCNMV), *Grapevine fan leaf virus* (GFLV), and *Potato virus X* (PVX) are all known to induce proliferation and invaginations of the ER (Ritzenthaler et al., 1995; Schaad et al., 1997; Carette et al., 2002; Lee and Ahlquist, 2003; Lee et al., 2003; Turner et al., 2004). The ER membranes serve as a scaffold for plant virus replication and movement complexes and/or they support virion maturation (Verchot, 2016).

Infection of *N. benthamiana* plants with *potato virus X* (PVX) induces a number of genes associated with the UPR including BIP, PDI, calreticulin (CRT) and calmodulin (CAM) (Ye et al., 2011). The viral component responsible for the activation has been traced to the triple gene block protein 3, TGBp3, a viral membrane movement protein. TGBp3 delivered on its own by a *tobacco mosaic virus* vector will also upregulate UPR-related factors. Not only does PVX upregulate the UPR, but the UPR helps to support PVX infection. This was revealed by silencing *N. benthamiana bZIP60* and finding that the silencing inhibits virus replication in protoplasts and delays virus systemic accumulation in plants.

The UPR also supports *turnip mosaic virus* (TuMV) infections demonstrated by the fact that double *ire1a ire1b* mutant suppresses TuMV symptoms in *Arabidopsis*. Since the major splicing target for IRE1a and IRE1b is bZIP60 mRNA, Zhang et al. (2015) reported that a knockout in bZIP60 also suppressed viral symptoms and that the suppression of symptoms in the bZIP60 knockout could be overcome by the transgenic expression of an activated form of bZIP60. They further showed that bZIP60 is spliced in response to virus infection and that a viral membrane protein, 6K2, on its own could elicit bZIP60 mRNA splicing in a *N. benthamiana* transient expression system (Zhang et al., 2015).

How could it be that TGBp3 in PVX and 6K2 in TuMV elicit the UPR? There is precedence for the expression of certain proteins causing ER stress. In *Arabidopsis* chronically misfolded forms of carboxypeptidase Y, CPY^∗^ (Finger et al., 1993) and zeolin, a fusion between two storage proteins, zein and phaseolin (Mainieri et al., 2004) produce ER-stress induced autophagy (autophagy that can be reversed by chemical chaperones) (Yang et al., 2016). Thus, TGBp3 in PVX and 6K2 in TuMV may be interpreted by the ERQC system as chronically misfolded proteins or they may interfere with the folding of other proteins.

The virulence determinant in *soybean mosaic virus* (SMV), a potyvirus, also elicits UPR in its host, but appears to do so by a different mode (Luan et al., 2016). The virulence factor in the potyvirus is the P3 protein, which is involved in a variety of functions including viral replication, movement and pathogenesis. Luan et al. (2016) showed that SMV P3 interacts with soybean translation elongation factor 1A (eEF1A). Using VIGs, the authors knocked down the expression of eEF1A, which reduced the ability of the plants to induce ER stress and rendered the plants more resistant to SMV.

## Conclusion

The UPR, which has been long associated with stress, also functions during normal development, defense and viral infection (**Figure 2B**). The conditions that elicit the response and the consequence of its induction are current subjects of investigation.

## Author Contributions

All authors listed, have made substantial, direct and intellectual contribution to the work, and approved it for publication.

## Conflict of Interest Statement

The authors declare that the research was conducted in the absence of any commercial or financial relationships that could be construed as a potential conflict of interest.
